# A novel near-infrared fluorescent probe for rapid sensing of HClO in living cells and zebrafish

**DOI:** 10.3389/fchem.2022.1009186

**Published:** 2022-09-21

**Authors:** Liangliang Li, Xiaofeng Wang, Jinzhi Huang, Kaidong Ma, Xiaoyu Tan

**Affiliations:** ^1^ Shenzhen Longhua District Central Hospital, Guangzhou, China; ^2^ Department of Otolaryngology-Head and Neck Surgery, The First Affiliated Hospital of Hainan Medical University, Haikou, China; ^3^ Shunde Women and Children’s Hospital of Guangdong Medical University, Foshan, Guangdong, China; ^4^ Shenzhen Longgang District Maternal and Child Health Hospital, Guangzhou, China

**Keywords:** fluorescent probe, HClO, near-infrared, living cells, zebrafish

## Abstract

Reactive oxygen species (ROS) are significant active species in living organisms, and their coordination maintains the function of organelles to resist the invasion of foreign substances. Hypochlorous acid (HClO) is not only an eventful signaling species but also a kind of ROS, which plays an irreplaceable role in the immune system. However, its abnormal levels can cause cell damage or even apoptosis, which in turn leads to the onset of a series of diseases such as inflammation, neurological diseases, and even cancer. Based on this, we designed a near-infrared fluorescent probe with a large Stokes shift for ultrafast response to HClO. Furthermore, the probe exhibits excellent sensitivity and selectivity toward HClO over other species. The probe was successfully applied to visualize endogenous and exogenous HClO in living cells and in zebrafish. This unique study is the key to providing a trustworthy tool for imaging based on the *in vitro* and *in vivo* imaging of endogenous HClO, which possesses great potential for the use in future studies of HClO-related biology and pathology.

## Introduction

Talking about the factors of living organisms, reactive oxygen species (ROS) are significant molecules that play a critical role in cellular homeostasis and information transfer. (Valko et al., 2007; Dickinson and Chang, 2011; Yang et al., 2019). It fights viruses and bacteria from invading the immune system, thereby protecting the human body from harm (Nicodeme et al., 2010; Prokopowicz et al., 2010; Lu et al., 2022). Hypochlorous acid (HClO) is considered to be a highly oxidative ROS, that has attracted much attention due to its important antibacterial properties in living organisms (Hampton et al., 1998; Fan et al., 2022). The oxidation of chloride ions in neutrophils by overexpressed myeloperoxidase produces HClO (Li et al., 2012). Although it is closely linked to cell metabolism, abnormal amounts can lead to rheumatoid arthritis, cardiovascular illness, neurological disease, and other conditions (Winterbourn and Kettle, 2000; Pattison and Davies, 2001; Fang, 2004). Furthermore, HClO is the main component of bleaching powder, and washing hands with it can reduce gynecological infection and maternal fever. Thus, it is considered to be a double-edged sword in biological systems (Kantarci et al., 2000). Therefore, it is necessary to develop a reliable analytical method to detect fluctuations in the level of HClO in order to study its relationship with related diseases and its mechanism of action.

Over the decades, for the detection of HClO, a variety of approaches have been developed, including high-performance liquid chromatography, electrochemical analysis, chemiluminescence, and luminescent/fluorescent methods. (Li et al., 2018; Yan et al., 2019; Luo P. et al., 2021). Fluorescence imaging combined with the small molecule method has received extensive attention because of its great spatiotemporal resolution, *in situ* monitoring, and ease of visualization (Kobayashi et al., 2010; Kumar et al., 2015; Zhang et al., 2020; Luo X. et al., 2021; Cheng et al., 2021). Therefore, it is possible to monitor individual compounds in biological systems in real time by fluorescence imaging. Currently, fluorescent probes for HClO have been developed for both *in vitro* and *in vivo* imaging (Zhou et al., 2012; Mao et al., 2018; Zhang et al., 2018; Ren et al., 2019; Wei et al., 2019; Wu et al., 2019; Lin et al., 2020; Shi et al., 2020; Xu et al., 2020; Fang et al., 2022). Unfortunately, there are more or less certain defects for some developed probes, including a slow reaction rate, short emission wavelength, and a small Stokes shift, which limit their capabilities for imaging and detecting HClO in living cells. Near-infrared (NIR) fluorescence dye displays particular virtues with deeper tissue penetration, minimum photodamage, and low background interference, which facilitates its application in biological systems (Yuan et al., 2013; Bonacchi et al., 2016; Hong et al., 2017; Luo et al., 2020; Wang et al., 2022). Furthermore, strong Stokes shift can be affective and preventive toward emission and excitation bands. As a result, developing a suitable fluorescence probe with a significant Stokes shift and NIR emission wavelength for imaging and detecting HClO in live systems is critical, which would be helpful for understanding the relationship between HClO and inflammation.

Herein, an easily obtained NIR fluorescent probe was proposed for the imaging and monitoring of HClO in a physiological environment. Initially, the probe emits negative fluorescence, while *N*, *N*-dimethylthiocarbamate is separated, and the probe releases significant NIR fluorescence upon reaction with HClO. Importantly, the probe responds quickly (10 s) and has a strong selectivity for HClO over other ROS molecules. The probe provides a large Stokes shift, avoiding crosstalk between the excitation and emission spectra in the existence of HClO. Furthermore, the probe was successfully used to detect HClO in living cells and zebrafish under oxidative stress conditions. We believe this probe shows powerful potential for imaging and understanding the relationship between HClO and inflammatory diseases.

## Section of experiments

### Instruments and reagents

Sigma-Aldrich provided cyclohexanone, propylene glycol, phosphorus tribromide (PBr_3_), cesium carbonate (Cs_2_CO_3_), 4-methylsalicylaldehyde, N, N-dimethylthiocarbamoyl chloride, boron tribromide (BBr_3_), 4-fluoro-2-hydroxybenzaldehyde, piperidine, and 4-chlorosalicylalde (St. Louis, United States). Macklin supplied lipopolysaccharide (LPS), uric acid (UA), and aminoguanidine hydrochloride (AG) (Shanghai, China). A Bruker Avance II NMR spectrometer was used to acquire ^1^H and ^13^C NMR spectra (Germany). The UV–vis and fluorescence spectra were collected while correlating with the with the F-7000 spectrophotometer (Japan). Moreover, the images were observed with the Olympus FV1000 microscope (Japan).

### Fluorescence detection for hypochlorous acid

In DMSO, a stock solution of the NIR fluorescent probe HDCX-HClO (1 mM) was produced. Other stock analyte (10 mM) solutions of amino acids, ROS/RNS, various anions and cations such as NO_2_^−^, H_2_O_2_, HNO, ^*t*^BuOO^.^, NO, ONOO^−^, and OH, common anions such as S_2_O_8_^2-^, C_2_O_4_^2-^, S_2_O_7_^2-^, HSO_4_^−^, SO_4_^2-^, CO_3_^2-^, HS^−^, NO_3_^−^, HCO_3_^−^, AcO^−^, HSO_3_^−^, F^−^, Cl^−^, and Br^−^, metal ions such as Ba^2+^, Hg^2+^, Mg^2+^, Fe^2+^, Fe^3+^, Cu^2+^, and Zn^2+^, and amino acids and biothiols such as methionine (Met), tryptophan (Trp), valine (Val), serine (Ser), lysine (Lys), aspartic acid (Asp), threonine (Thr), alanine (Ala), arginine (Arg), and isoleucine (Ile) were prepared in ultra-pure water. All the spectral experiments were carried out at physiological pH.

### Fluorescence imaging in living cells and zebrafish

RAW 264.7 cells were grown in DMEM with 10% FBS and penicillin (100 units/mL)-streptomycin (100 g/ml) liquid. The cells were placed in a 95 % environment with 5% CO_2_ at 37°C. The cytotoxicity of the probe was determined *via* the Cell Counting Kit-8 (CCK-8) test. The fluorescent image was recorded on the Olympus FV1000 microscope. Before imaging experiments, the cells were seeded in a culture dish and then incubated for 24 h. After washing with PBS, the cells were stained with the probe (10 μM) and further incubated for 20 min.

The zebrafish was cultured with E3 embryo medium at around 28.5°C. For control, 4-day-old zebrafish was incubated in E3 embryo medium and stained by HDCX-HClO for 30 min and then washed with the culture medium before imaging experiments. For imaging exogenous and endogenous HClO in zebrafish, 4-day-old zebrafish was treated in E3 embryo medium containing HClO or LPS for 10 min or 12 h and then cultured with the probe HDCX-HClO for 30 min, respectively. These zebrafish were washed with the medium three times and then mounted on a microscope stage. Confocal fluorescence emission collection window: 690–770 nm.

### Synthesis of compound HDCM-hypochlorous acid

Compounds HDCX-OH (0.418 g, 1 mmol) and N, N-dimethylthiocarbamoyl chloride (0.25 g, 2 mmol) were added in a three-necked flask with 10 ml anhydrous ethanol. The mixture was vigorously agitated and refluxed for approximately 12 h in a N_2_ atmosphere. ^1^H NMR (500 MHz, CDCl_3_) δ (ppm) 8.09 (d, *J* = 15.5 Hz, 1H), 7.77–7.68 (m, 1H), 7.62 (d, *J* = 8.1 Hz, 1H), 7.48–7.36 (m, 2H), 7.22–7.10 (m, 2H), 6.89 (d, *J* = 8.2 Hz, 1H), 6.78 (d, *J* = 4.2 Hz, 2H), 6.52 (s, 1H), 3.48 (s, 3H), 3.40 (s, 3H), 2.62–2.57 (m, 2H), 2.51–2.46 (m, 2H), and 2.03 (d, *J* = 5.4 Hz, 2H). ^13^C NMR (125 MHz, CDCl_3_) δ (ppm) 161.7, 153.2, 152.9, 134.6, 133.7, 130.9, 128.8, 128.7, 126.1, 123.7, 118.7, 116.6, 115.5, 114.4, 111.4, 110.4, 105.5, 68.2, 45.0, 44.1, 31.4, 29.7, and 20.5. HR-MS: calcd for C_30_H_23_N_3_O_3_S^+^, 505.1460; found, [M + Na]^+^, 528.1364.

## Results and discussion

### Probe synthesis and designing the rationale

**FIGURE 1:**
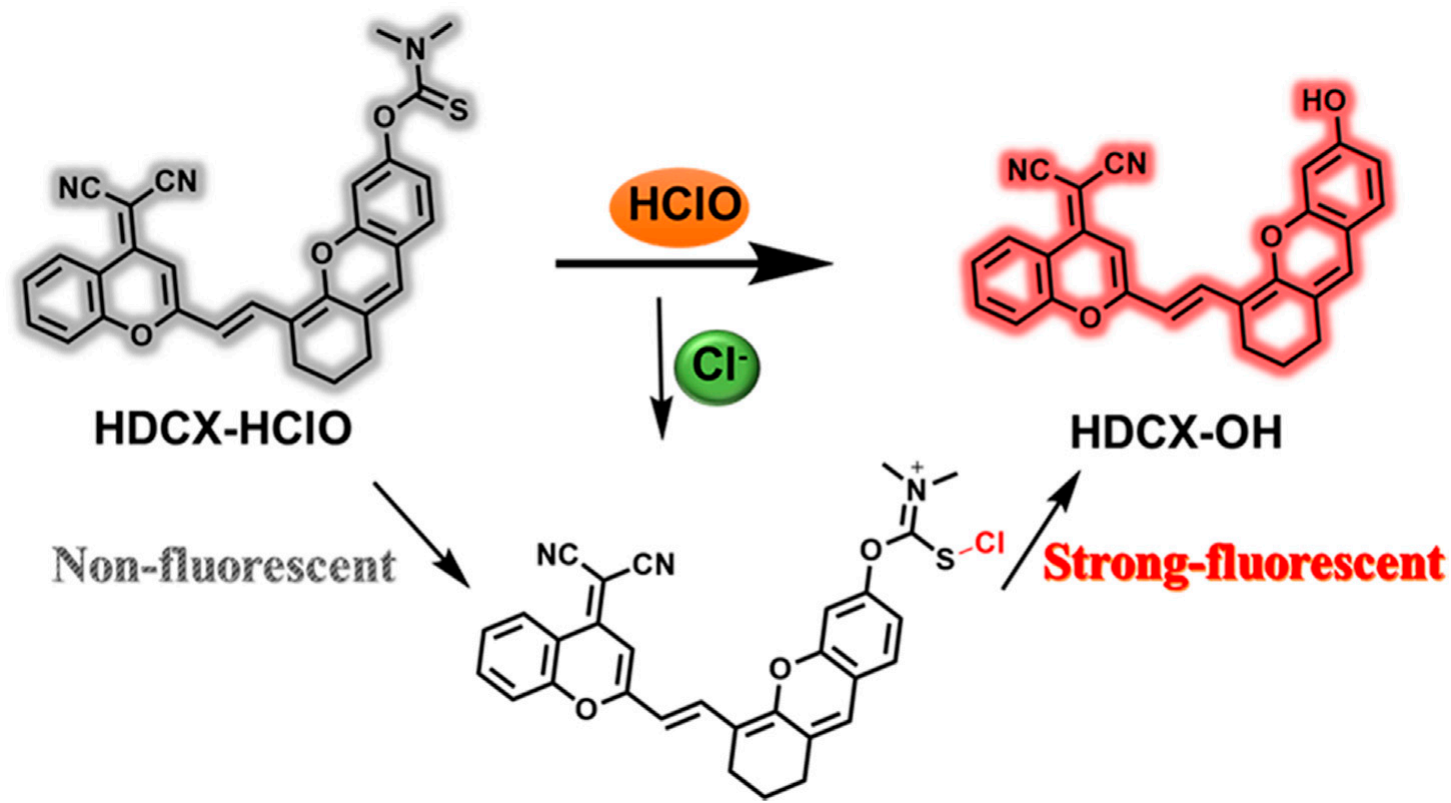
Sensing mechanism of HDCX-HClO to HClO.

**FIGURE 2:**
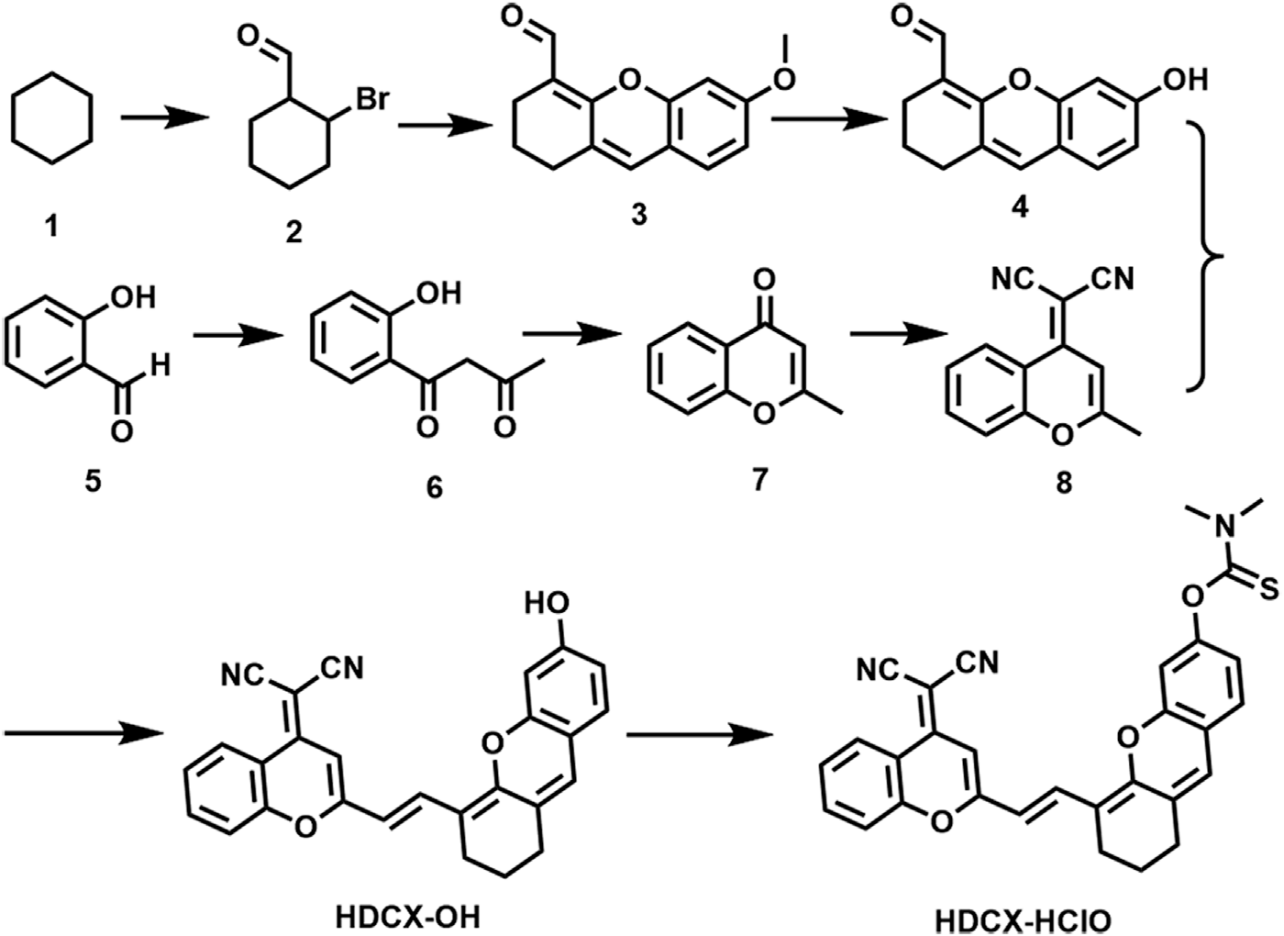
Synthesis route of compound HDCX-HClO.

We committed to developing a large Stokes shift and near-infrared fluorescence probe (HDCX-HClO, *Φ* = 2.1%) to monitor and image HClO in biological systems. The chemical structure and proposed reaction mechanism of HDCX-HClO toward HClO are illustrated in Figure 1. We selected HDCX-OH (*Φ* = 13%) as a fluorophore that is composed of electron-withdrawing and electron-donating groups. It showed a near-infrared emission peak and a significant Stokes shift (Qi et al., 2017). Furthermore, several reports reveal that N, N-dimethylthiocarbamate acts as an excellent acceptor of HClO in its place of other ROS. We guessed that the introduction of N, N-dimethylthiocarbamate would render HDCX-HClO non-fluorescent. However, in the presence of HClO, the hydroxyl group of the probe was released, which in turn exhibited a dramatic NIR fluorescence emission. Moreover, mass spectrometry analysis was further performed to prove the proposed response mechanism. As shown in Supplementary Figure S1, for HDCX-HClO, the peak was at m/z = 528.1345. However, a new peak at m/z = 441.1229 was observed, and the peak at m/z = 528.1345 (corresponding to HDCX-OH) declines in the presence of ONOO^−^ (Supplementary Figure S1). Meanwhile, the proposed mechanism was verified by DFT theoretical calculations (HOMO and LUMO orbitals of HDCX-OH and HDCX-HClO ) (Supplementary Figure S2). Motivated by this rationale, HDCX-HClO was synthesized, and the detailed structure and route of the goal substance are outlined in Figure 2. Furthermore, the characterization of the probe (^1^H NMR, ^13^C NMR, and HR-MS) was described accordingly.

### Spectroscopic properties

**FIGURE 3:**
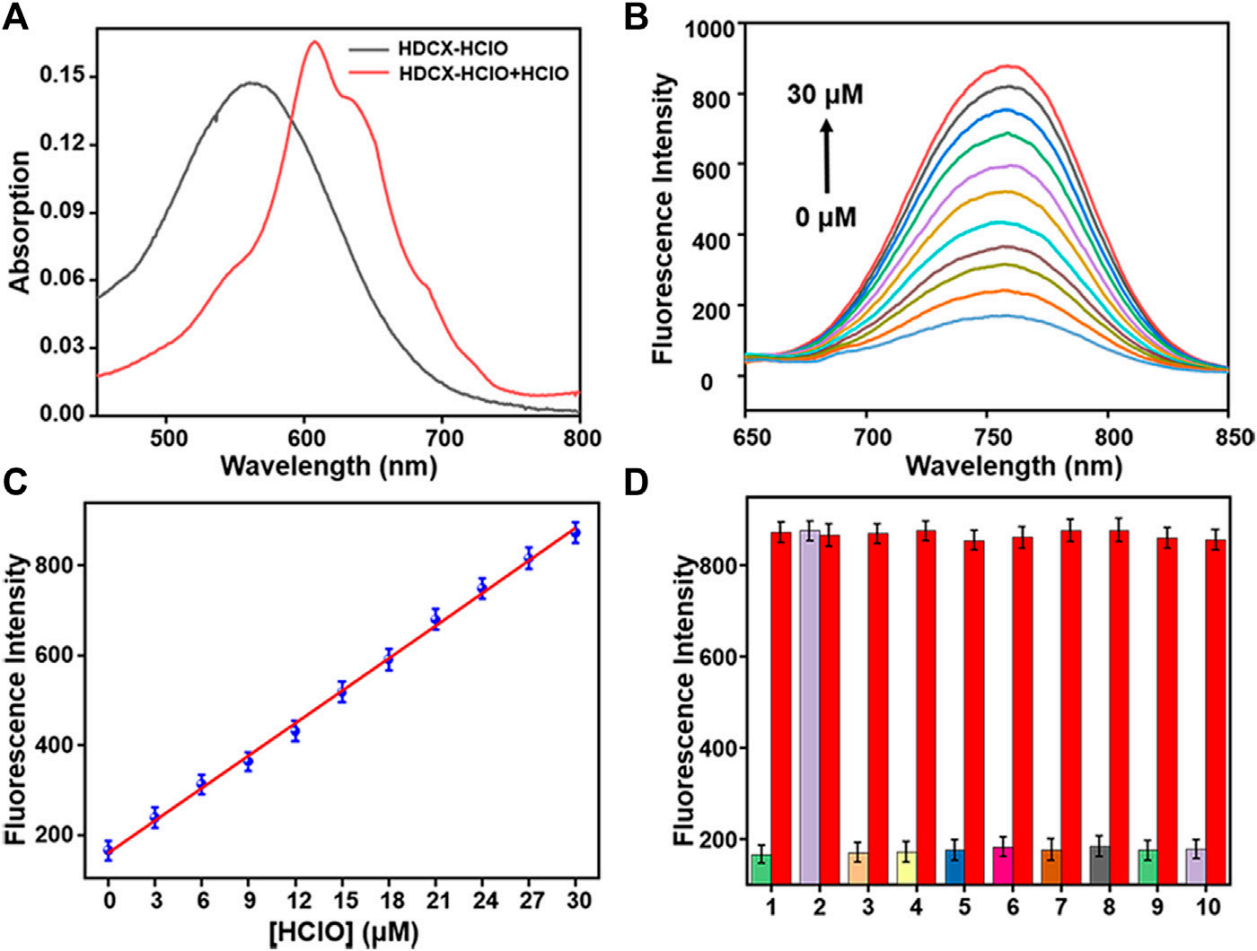
**(A)** Absorption spectra of HDCX-HClO (10 μM) in presence or absence HClO (30 μM). **(B)** Fluorescence spectra of HDCX-HClO toward various levels of HClO (0–30 μM). **(C)** Probe HDCX-HClO versus HClO concentrations. **(D)** Fluorescence intensity of HDCX-HClO (10 μM) for other analytes: 1. blank; 2. HClO; 3. NO_2_^−^; 4. ·OH; 5. ^*t*^BuOO^.^; 6. ONOO^−^; 7. NO; 8. H_2_O_2_; 9. HNO; 10. O_2_·^-^. The spectrum was obtained in PBS solution containing 40% DMSO (10 mM, pH 7.4) at room temperature. λ_ex_ = 590 nm.

**FIGURE 4:**
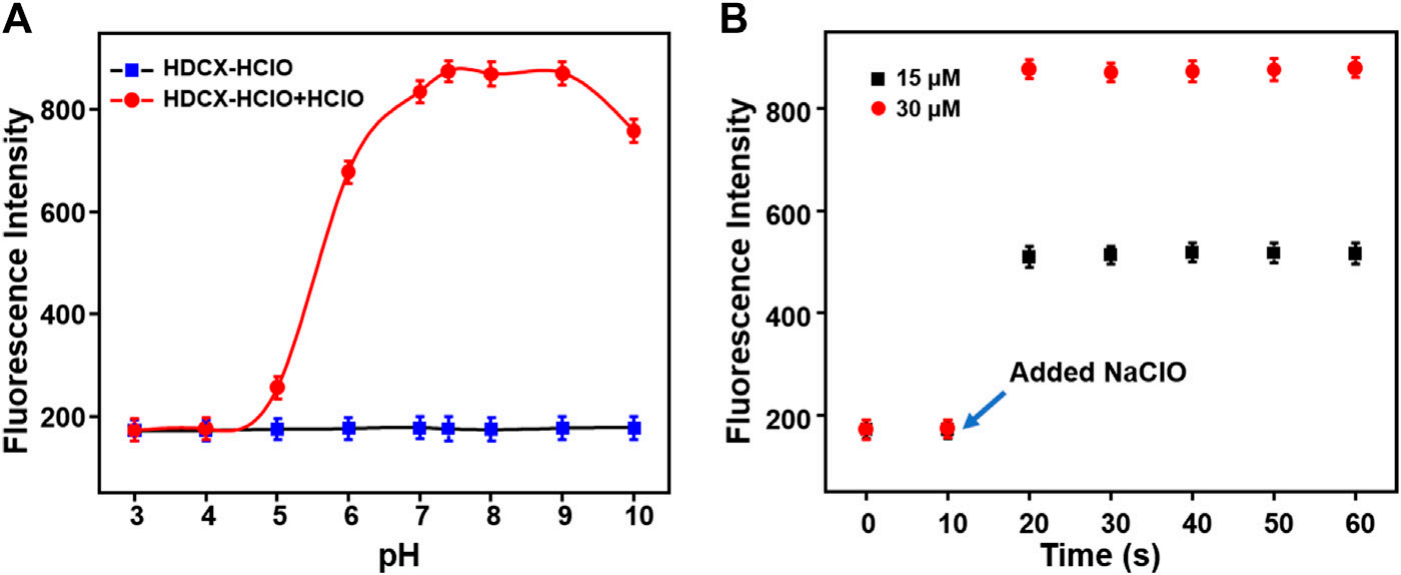
**(A)** Response rate and **(B)** pH effect of HDCX-HClO for HClO.

It has been observed that the absorption and fluorescence spectral peculiarities of HDCX-HClO in the presence or absence of HClO were discretely investigated in 10 mM PBS buffer solution (pH 7.4, with 40% DMSO). As depicted in Figure 3A, the probe HDCX-HClO displayed an absorbance at around 560 nm. Upon reaction with HClO, there was a red shift of the absorption maximum to center at 605 nm. It should have the emergence of intramolecular charge transfer (ICT) and further release the initial fluorophore (HDCX-HClO). Furthermore, the fluorescence titration experiment of HDCX-HClO toward HClO was performed. As we expected, the free HDCX-HClO had a negligible fluorescence signal at 750 nm. However, significant fluorescence intensity was found to be concentrated at 750 nm after the addition of HClO, which would be attributed to the specific response of the probe’s responsive group N, N-dimethylthiocarbamate to HClO and further caused the release of the fluorophore (HDCX-OH). Notably, a significant Stokes shift (>100 nm) was observed, which is beneficial for reducing self-quenching. The fluorescence of HDCX-HClO enhanced with the contents of HClO gradually increased in the range of 0–30 μM (Figure 3B). The HDCX-HClO had a great linear relation with the levels of HClO ranging from 0 to 30 μM (Figure 3C). The associated regression equation was fitted to F_750 nm_ = 160.6417 + 24.0578 (HClO) and (*R* ^2^ = 0.9881). Furthermore, the detection limit (LOD, 3σ/k) for HClO was determined to be 26 nM. These results revealed that the probe HDCX-HClO could quantitatively monitor HClO with high sensitivity and shows potential to be applied to trace amounts of HClO in cells. Subsequently, the pH (3–10) on the fluorescence signal of the probe HDCX-HClO for HOCl was assessed in the absence or presence of HOCl. In Figure 4A, the probe HDCX-HClO was largely unaffected by pH, while the probe displayed excellent fluorescence intensity when the pH ranged from 7–9 under the presence of HClO. The results indicated that HDCX-HClO could sensitively detect HClO under physiological conditions and be employed to image HClO in biological systems, principally.

Next, the response rates of HDCX-HClO toward HClO were investigated *via* the fluorescence intensity change of HDCX-HClO at different levels of HClO in real time. As shown in Figure 4B, there was an apparent enhancement of fluorescence intensity in the presence of varied HClO, which reached the peak almost simultaneously within 10 s. In order to apply the probes to complex biological systems, we first verify whether the probes are sensitive to specific reactions to the detection substances. We studied the selectivity of the probe HDCX-HClO for HClO by recording the fluctuation of the fluorescence emission intensity. A series of analytes were evaluated, such as NO_2_^−^, ·OH, ^*t*^BuOO^.^, ONOO^−^, NO, H_2_O_2_, HNO, and O_2_·^-^, common anions such as S_2_O_8_^2-^, C_2_O_4_^2-^, S_2_O_7_^2-^, SO_3_^2-^, HSO_4_^−^, SO_4_^2-^, CO_3_^2-^, HS^−^, NO_3_^−^, HCO_3_^−^, AcO^−^, HSO_3_^−^, F^−^, Cl^−^, and Br^−^, metal ions such as Ba^2+^, Hg^2+^, Mg^2+^, Fe^2+^, Fe^3+^, Cu^2+^, and Zn^2+^, and amino acids and biothiols such as Met, Trp, Val, Phe, Glu, Ser, Lys, Asp, Thr, Ala, Arg, Ile, Tyr, GSH, Hcy, and Cys. As illustrated in Figure 3D, HClO could lead to significantly enhanced fluorescence signal, while negligible fluorescence intensity was observed for other species containing ROS/RNS. The result suggested that HDCX-HClO was highly selective for HClO. Furthermore, as depicted in Supplementary Figure S1, S2, even when these analytes co-existed with HClO, the probes still displayed strong fluorescence, which further demonstrated the powerful anti-interference ability of HDCX-HClO in response to HClO. Overall, the probe could quickly and specifically respond to HClO under physiological conditions, which may be applied for the detection of HClO in biological systems. In general, the probes exhibit great potential in the specific and rapid detection of HClO under physiological conditions.

### Fluorescence imaging of hypochlorous acid in living cells

**FIGURE 5:**
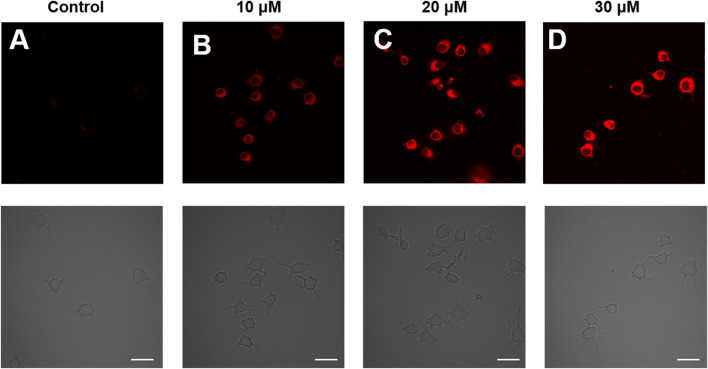
Imaging of HClO in RAW 264.7 cells using a confocal microscope with the HDCX-HClO probe (10 μM). **(A)** RAW 264.7 cells serve as the control group. Before imaging that is shown in **(B–D)**, the cells were pre-incubated within half an hour concerning HDCX-HClO and followed by the addition of HClO (10, 20, and 30 μM) for 10 min in each of the three groups. Fluorescent images were recorded with excitation at 561 nm and emission at 690–770 nm. Scale bar = 20 μm.

The HDCX-HClO probe was used to image HOCl in living systems and evaluate its ability to denote RAW 264.7 cell lines. Prior to the imaging applications of the probe, we evaluated the cytotoxicity of HDCX-HClO on RAW 264.7 cells by a CCK-8 assay. As shown in Supplementary Figure S3, the cell viability remained above 80% even after incubation with high concentrations of HDCX-HClO, demonstrating excellent biocompatibility with a clinically permissible dose of approximately 10 μM. With excellent sensitivity and low cytotoxicity, the probe can be used to detect the presence of HClO in living cells. We then investigated the feasibility of detecting cellular HClO using the probe HDCX-HClO. As illustrated in Figure 5A, the RAW 264.7 cells displayed a bright fluorescence signal when incubated with the probe. However, the HDCX-HClO-loaded RAW 264.7 cells concerning different statuses of HClO (10, 20, and 30 μM) for around 20 min, with significant fluorescence enhancement, were observed. Moreover, the probe exhibited a distinct fluorescence response for various levels of HClO with high sensitivity, which provided the possibility to visualize the quantitative detection of HClO in cells.

**FIGURE 6:**
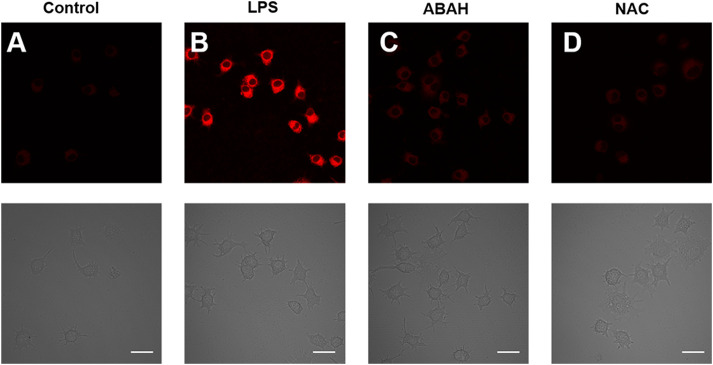
Fluorescence microscopic images of exogenous HClO with HDCX-HClO in RAW 264.7 cells. **(A)** Control group: The RAW 264.7 cells were incubated with the probe for 30 min before being imaged with the probe. **(B)** Cells were treated with LPS (100 ng/ml) and PMA (100 ng/ml) in a continuous fashion for 24 h before being treated with HDCX-HClO. LPS/PMA (100 ng/ml), **(C)** ABAH (200 ng/ml), **(D)** NAC (1 mM), and then incubated with HDCX-HClO were all used to treat the cells in this experiment and the 561 nm and emission at 690–770 nm correlated with it. Scale bar = 20 μm.

To further study the performance of HDCX-HClO for the monitoring of endogenous HClO, the potential imaging of HDCX-HClO was assessed *via* RAW 264.7 cells stimulated with lipopolysaccharide (LPS) and phorbol-12-myristate-13-acetate (PMA). The release of high levels of HClO in cells and animals under the treatment with LPS/PMA is well documented. (Gordon et al., 1987). First and foremost, there is a faint fluorescence signal after the RAW 264.7 cells were cultured with HDCX-HClO (Figure 6A). Then, the RAW 264.7 cells were treated with LPS/PMA, and it was discovered that there was a significant increase in the fluorescence signal. (Figure 6B). This was attributed to LPS/PMA-induced intracellular oxidative stress, resulting in an increase in endogenous HClO levels, suggesting that this probe could be applied to the detection of endogenous HClO in cells. 4-Aminobenzoic acid hydrazide (ABAH) was used to reduce the levels of HClO due to its ability to inhibit the activity of myeloperoxidase. As expected, when compared with the LPS/PMA group, signals obtained when RAW 264.7 cells were simultaneously incubated with ABAH and LPS/PMA were reduced, which suggested that the level of HClO decreased (Figure 6C). Similarly, the experimental phenomenon was negative (Figure 6D). All the aforementioned results clearly revealed that HDCX-HClO could be suitable for monitoring and imaging of the variations of endogenous and exogenous HClO in biological systems.

### Zebrafish and mapping hypochlorous acid

**FIGURE 7:**
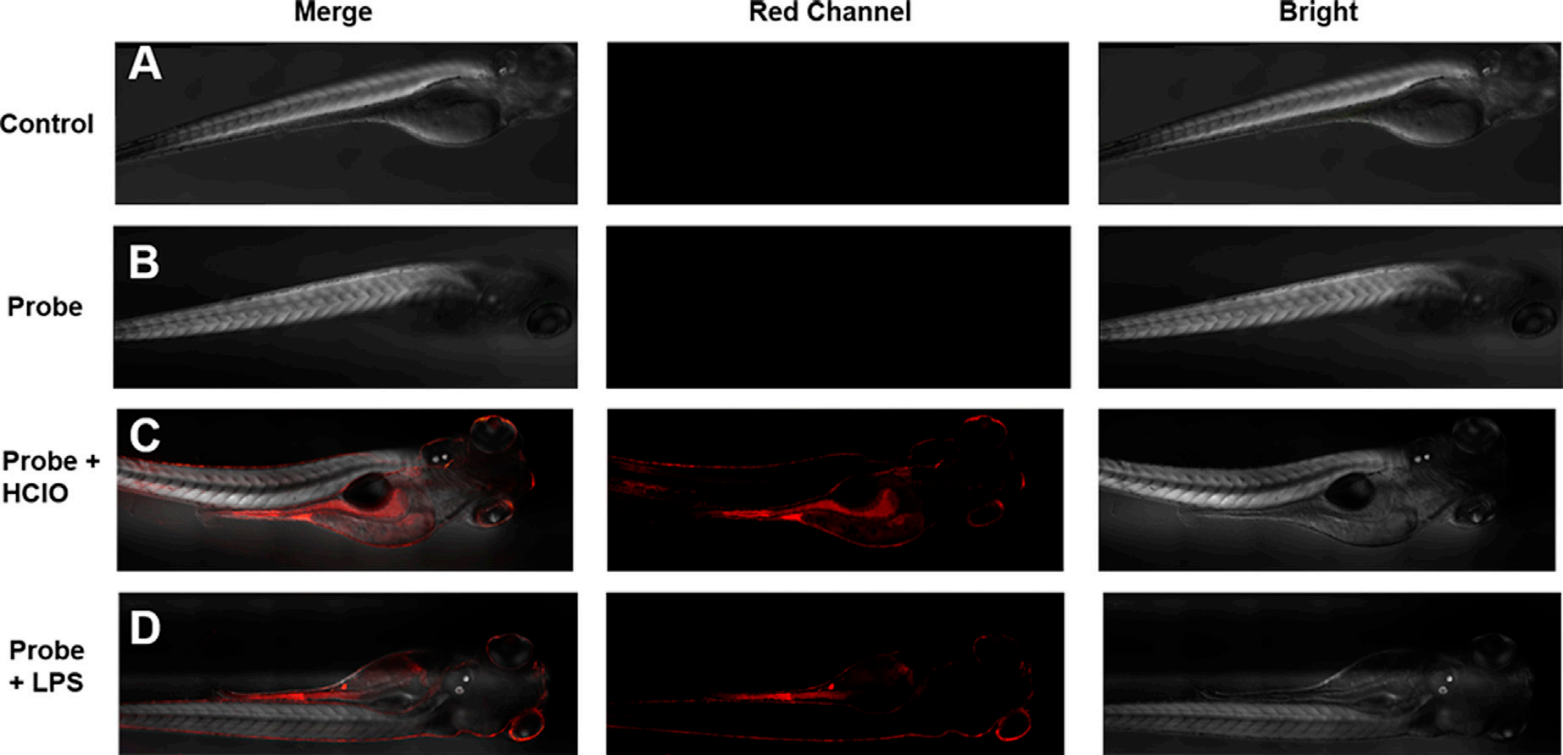
Imaging of HClO in zebrafish with the probe HDCX-HClO (10 μM). **(A)** Zebrafish blank without dealing with the probe; **(B)** zebrafish was incubated with HDCX-HClO for 30 min before imaging; **(C)** zebrafish loaded with the probe HDCX-HClO and 30 μM HClO; **(D)** zebrafish was stimulated with LPS/PMA and then stained by HDCX-HClO for 30 min before imaging.

In order to evaluate the biological application of the probe HDCX-HClO *in vivo*, we used a 4-day-old zebrafish as a research model to collect further data (Figure 7); the zebrafish was not dealing with any, and no fluorescence signal was observed. Subsequently, the zebrafish was stained with 10 μM probe HDCX-HClO for 30 min, and an inert fluorescence was found (Figure 7B). The zebrafish, on the other hand, was treated with HClO for 10 min after being incubated with HDCX-HClO for another 30 min. The fluorescence images showed a definite progression in color over time, which directly reveals fluctuations in HClO levels *in vivo* (Figure 7C). Interestingly, the fluorescence image result displayed HClO in the liver and intestine was relatively greater than that in the other organs when the zebrafish was treated with HClO. It could be attributed to the basic physiological functions of the liver and intestine to eliminate toxic substances (Hao et al., 2021). To discuss the feasibility of HDCX-HClO, the zebrafish was exposed with LPS in E3 embryo medium for about 6 h and stained with the probe. Eventually (Figure 7D), a light red fluorescence image was obtained that demonstrated the native HClO could be gauged *via* HDCX-HClO. Taken together, HDCX-HClO can be used as a tool to detect and image HClO both exogenously and endogenously *in vivo*.

## Conclusion

Overall, as concluding remarks, we developed a novel near-infrared fluorescent probe, HDCX-HClO, to track and image exogenous and endogenous HClO in living cells and zebrafish. In comparison to previous ROS/RNS, the probe exhibits greater specificity and sensitivity to HClO. Because of the ICT mechanism, there was a rapid increase in fluorescence within 10 s of the addition of HClO to the reaction mixture. The fact that HDCX-HClO has a low detection limit (26 nM) and is extremely stable is worth emphasizing as it provides a reliable foundation for the application of HClO in biological systems. Moreover, the probe HDCX-HClO was demonstrated for its ability to observe and monitor both exogenous and endogenous HClO in a variety of conditions. The probe can also detect alterations in HClO levels in living cells and zebrafish when they are subjected to LPS-induced oxidative stress, making it a helpful tool for studying the relationship between inflammatory illnesses and HClO levels in the body.

## Data Availability

The original contributions presented in the study are included in the article/Supplementary Material; further inquiries can be directed to the corresponding author.
