# Supplementary material for: A novel near-infrared fluorescent probe for rapid sensing of HClO in living cells and zebrafish
Source: Front Chem. 2022 Sep 21;10:1009186. doi: 10.3389/fchem.2022.1009186 (PMC9532538; doi:10.3389/fchem.2022.1009186)
Supplement: Supplementary file 1 [file DataSheet1.docx]

**A novel near infrared fluorescent probe for rapid sensing of HClO in living cells and** **zebrafish**

Liangliang Li†^1^, Xiaofeng Wang†^4^, Jinzhi Huang*^2^, Kaidong Ma^1^, Xiaoyu Tan^3^

1 Shenzhen Longhua District Central Hospital, Guangzhou, 518000, People’s Republic of China

2 Shunde Women and Children’s Hospital of Guangdong Medical University, Guangdong Province, People’s Republic of China

3 Shenzhen Longgang District Maternal and Child Health Hospital, Guangzhou, 518000, People’s Republic of China

4 Department of Otolaryngology-Head and Neck Surgery, the First Affiliated Hospital of Hainan Medical University, Haikou, 570102, China

*Correspondence to:

Jinzhi Huang, Email: huangjzgd@163.com

**1. Synthesis of compounds**

**Synthesis of compound 2.**

9 mL of DMF to 50 mL of CH_2_Cl_2_ were added in a three-necked flask and then stirred at 0 °C for 10 min. Next, 10 mL of phosphorus tribromide were added dropwise to the mixture. After stirring for 60 min, 4 mL of cyclohexanone was added dropwise and stirred vigorously for overnight at room temperature. Adjusted the pH to neutral, extracted with CH_2_Cl_2_, dried, and directly put into the next step without purification.

**Synthesis of compound 3.**

Compound **2** (1.89 g, 10 mmol), Cs_2_CO_3_ (6.5 g, 2 mmol) and 2-Hydroxy-4-methoxybenzaldehyde (1.52 g, 10 mmol) were added in a round bottom flask. This mixture was dissolved in 30 mL of dry DMF and stirred at room temperature for 18 h. Water was added to quench the reaction and extracted with CH_2_Cl_2_. The organic phase was then dried with anhydrous Na_2_SO_4_ and the solvent was evaporation under reduced press. Removed the solvent, the obtained crude product was performed on silica column chromatography with petroleum ether/CH_2_Cl_2_ (3/1, v/v) to give yellow solids. Yield: 136 mg (62%). ^1^H NMR (500 MHz, d_6_-DMSO) δ 10.25 (s, 1H), 7.30 (d, *J* = 8.5 Hz, 1H), 6.98 (s, 1H), 6.90 (s, 1H), 6.77 (d, *J* = 8.5 Hz, 1H), 3.81 (s, 3H), 2.58 – 2.54 (d, *J* = 12.0 Hz, 2H), 2.30 (t, *J* = 6.0 Hz, 2H), 1.63 (dt, *J* = 12.0, 6.0 Hz, 2H).

**Synthesis of compound 4.**

Compound **3** (1.21 g, 5 mmol) was dissolved in 20 mL dry CH_2_Cl_2_, then BBr_3_ (1.5 mL, 15 mmol) was dropwise added into the mixture at 0 °C and reacted overnight at room temperature. It was put into ice-water and adjusted the pH to neutral. Extraction with CH_2_Cl_2_ and removal of the solvent under reduced pressure. The obtained crude product was performed on silica column chromatography with CH_2_Cl_2_/CH_2_OH (100/1, v/v) to give yellow solids. Yield: 73 mg (54%). ^1^H NMR (500 MHz, d_6_-DMSO) δ 10.20 (s, 1H), 10.19 (s, 1H), 7.20 (d, *J* = 8.3 Hz, 1H), 6.93 (s, 1H), 6.64 (s, 1H), 6.61 (d, *J* = 8.3 Hz, 1H), 2.57 – 2.52 (d, *J* = 12.0 Hz, 2H), 2.29 (t, *J* = 6.0 Hz, 2H), 1.65 – 1.59 (m, 2H).

**Synthesis of compound 6**. 2-Hydroxybenzaldehyde (2.5 g, 20 mmol) was added to a round bottom flask containing 30 mL of ethyl acetate. Then sodium metal (2 g, 87 mmol) was added and stirred at room temperature for 18 h. After the substitution reaction was completed, 3 mL of MeOH was added to completely react the sodium metal. Then 50 mL of HCl (1M) was added dropwise, and extracted with ethyl acetate, the organic phase was dried over Na_2_SO_4_. After removing the solvent, compound 1 was obtained as a yellow solid, which was directly processed in the next step without treatment.

**Synthesis of compound 7**. Compound 1 and 1 mL HCl was added to a round-bottom flask containing 50 mL MeOH, and heated to reflux for 4 h. Then the solvent was removed, ethyl acetate was added, and washed with saturated NaCl aqueous solution until colorless. After removing the solvent, the residues were purified by silica column chromatography (200 - 300 mesh) with a gradient eluent of petroleum ether and ethyl acetate (10 : 1, v:v) to give a white solid (2.66 g, yield: 41%). 1H NMR (400 MHz, CD_3_OD) δ(ppm): 8.15 - 8.17 (m, 1H), 7.60-7.64 (m, 1H), 7.36-7.41 (m, 2H), 6.16-6.17 (s, 1H), 2.37 (s, 3H).

**Synthesis of compound 8**. Compound 2 (2.4 g, 15 mmol) and malononitrile (1.98 g, 30 mmol) were dissolved in 15 mL of acetic anhydride. The mix was refluxed for 14 h and then the solvent was evaporated in vacuo. Deionized water (80 mL) was added to the residue, refluxed for 0.5 h, and then extracted with dichloromethane. The organic layer was dried on Na_2_SO_4_, filtered and concentrated. The resulting crude product was purified by silica gel column chromatography with gradient eluent of CH_2_Cl_2_ to obtain an orange solid (1.22 g, yield: 41%). ^1^H NMR (400 MHz, CD_3_OD) δ(ppm): 8.88-8.91 (m, 1H), 7.69-7.71 (m, 1H), 7.44-7.46 (m, 2H), 6.70 (s, 1H), 2.43 (s, 3H).

**Synthesis of compound** **HDCM-OH.** Under the protection of N_2_, compound 4 (0.23 g, 1 mmol) and compound 8 (0.21 g, 1mmol) were added to 10 mL of anhydrous ethanol. This mixture was stirred and refluxed for 12 h and filtered to obtain solid. The obtained crude product was purified on silica gel chromatography (200 - 300 mesh) with gradient eluent of CH_2_Cl_2_ to obtain compound. ^1^H NMR (400 MHz, CDCl3) δ 8.89 (d, J = 8.3 Hz, 1H), 8.09 (d, J = 15.4 Hz, 1H), 7.88 (d, J = 7.8 Hz, 2H), 7.74 (t, J = 7.8 Hz, 1H), 7.53 (d, J = 8.3 Hz, 1H), 7.47 (d, J = 7.8 Hz, 2H), 7.40 (t, J = 7.7 Hz, 1H), 7.02 (d, J = 8.3 Hz, 1H), 6.72-6.68 (m, 3H), 6.48 (s, 1H), 6.06 (d, J = 15.4 Hz, 1H), 5.16 (s, 2H), 2.55 (t, J = 5.6 Hz, 2H), 2.46 (t, J = 5.6 Hz, 2H), 1.86-1.81 (m, 2H), 1.35 (s, 12H). ^13^C NMR (100 MHz, CDCl3, Fig. S5) δ 160.2, 159.4, 153.9, 152.9, 152.5, 139.5, 135.15, 134.1, 127.6, 127.1, 126.5, 125.8, 125.5, 124.5, 118.5, 118.1, 115.7, 113.3, 110.6, 105.5, 101.9, 83.9, 70.3, 59.3, 31.9, 29.7, 24.9, 24.5, 22.7, 20.7.

**2. Mass spectra to verify proposed mechanism**

**
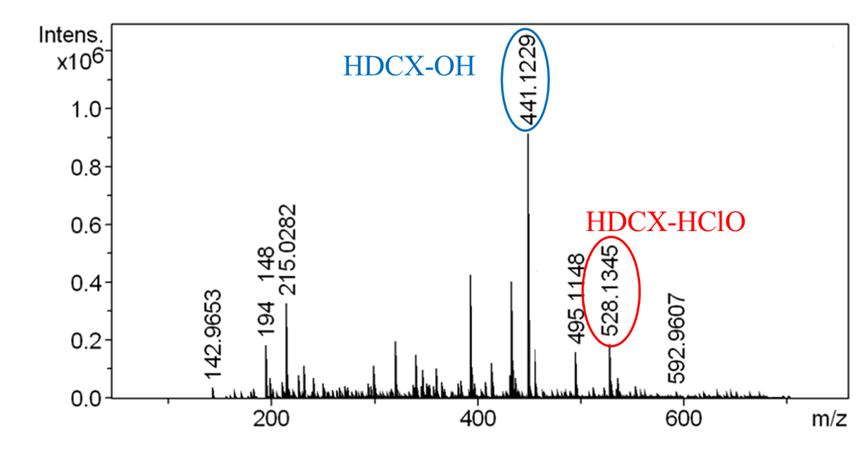
**

**Figure S1.** Mass spectra to verify proposed mechanism.

**3. HOMO and LUMO orbitals of** **HDCX-OH and** **HDCX-HClO.**

The HOMO and LUMO of HDCX-OH and HDCX-HClO were given in Figure S1. The HOMO-LUMO energy gaps of HDCX-OH and HDCX-HClO are calculated as 2.50 and 2.40 eV, respectively. HDCX-OH is composed of the donor (push) linking with an acceptor (pull), which enables an ICT from the donor to the acceptor to form a push-pull system and makes the fluorescence recover. On the contrary, the N, N-dimethylthiocarbamoyl ester group of HDCX-HClO blocks the intramolecular ICT effect, which causes the fluorescence be quenched. The theoretical calculations are in accordance with the experimental conclusions, which confirm the proposed reaction mechanism.


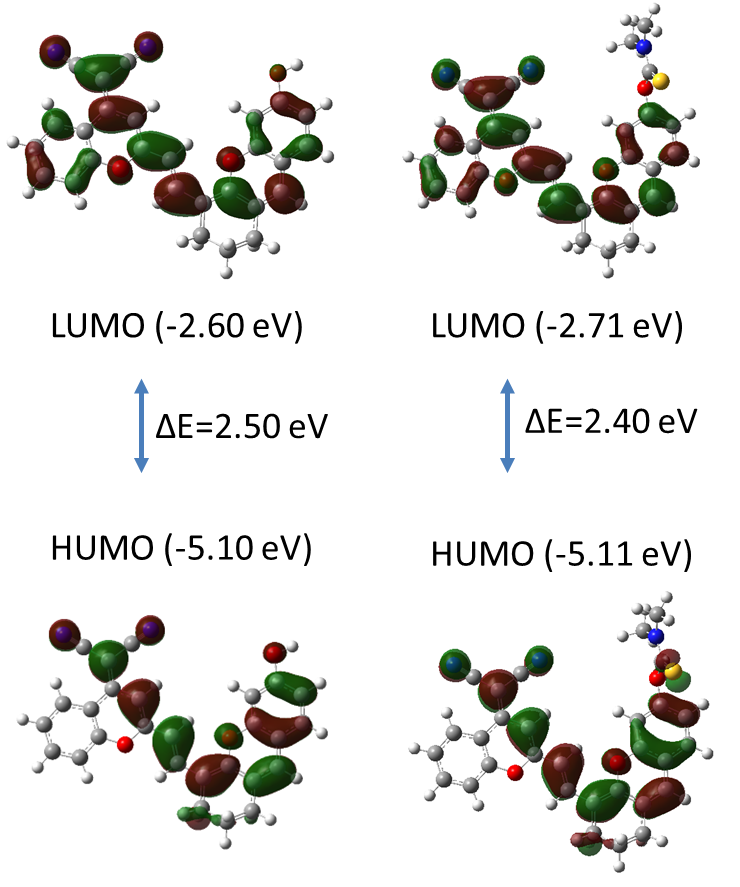


**Figure S2.** DFT optimized molecular orbital plots (LUMO and HOMO) of HDCX-OH and HDCX-HClO.

**4. Fluorescence spectra of HDCX-HClO toward HClO.**


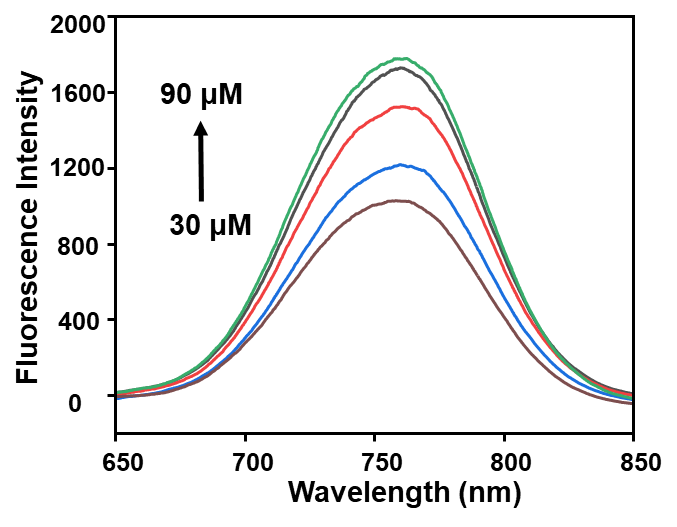


**Figure S3.** Fluorescence spectra of HDCX-HClO toward various levels of HClO (30-90 μM).

**5. Selectivity of HDCX-HClO.**


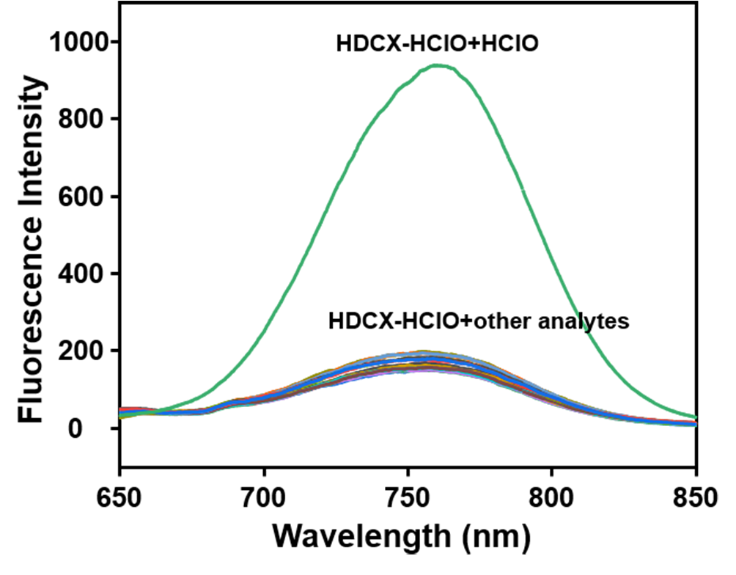


**Figure S4.** Fluorescence spectroscopy of the probe HDCX-HClO to HClO and other analytes.


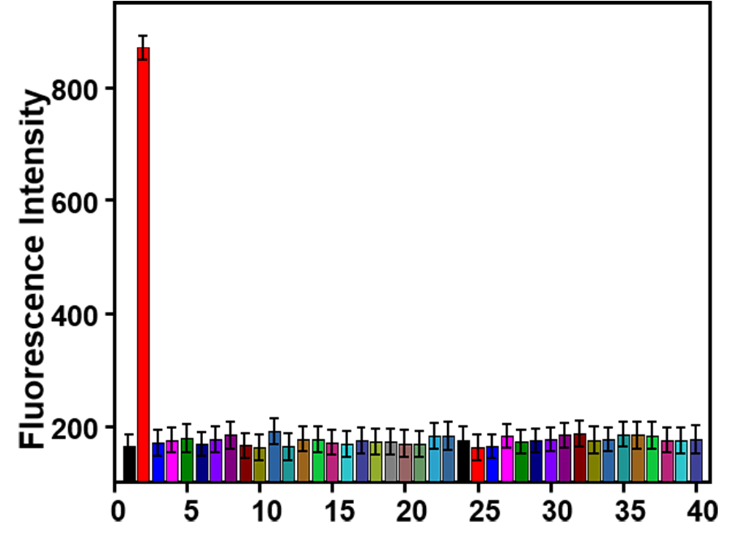


**Figure S5.** Fluorescence intensity of the probe HDCX-HClO to HClO and other analytes. 1. Blank, 2. HClO, 3. S_2_O_8_^2-^, 4. C_2_O_4_^2-^, 5. S_2_O_7_^2-^, 6. SO_3_^2-^, 7. HSO_4_^-^, 8. SO_4_^2-^, 9. CO_3_^2-^, 10. HS^-^, 11. NO_3_^-^, 12. HCO_3_^-^, 13. AcO^-^, 14. HSO_3_^-^, 15. F^-^, 16. Cl^-^, 17. Br^-^, 18. Ba^2+^, 19. Hg^2+^, 20. Mg^2+^, 21. Fe^2+^, 22. Fe^3+^, 23. Cu^2+^, 24. Zn^2+^, 25. Met, 26. Trp, 27. Val, 28. Phe, 29. Glu, 30. Ser, 31. Lys, 32. Asp, 33. Thr, 34. Ala, 35. Arg, 36. Ile, 37. Tyr, 38. Cys, 39. GSH, 40. Hcy.

**6. Cytotoxicity assays**


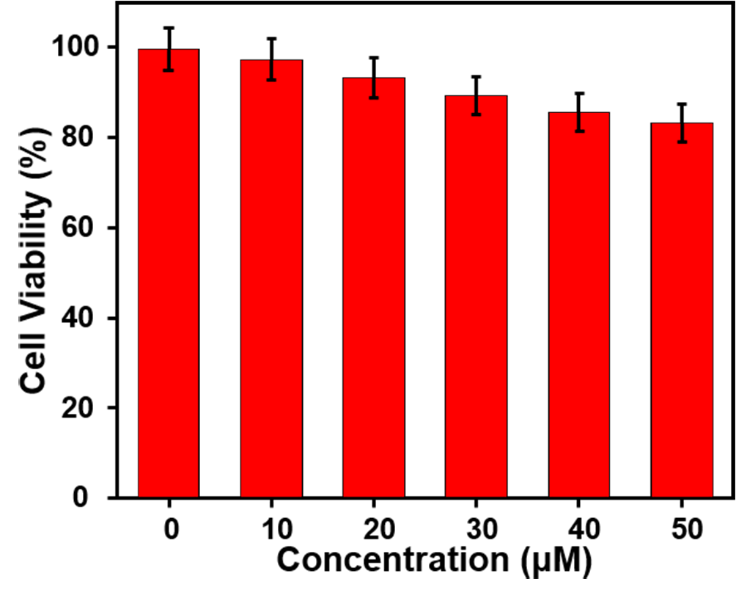


**Figure S6.** The cell viability of the probe against RAW 264.7 cells. The experiments were repeated three times and the data were shown as mean (± S.D.).

**7. ^1^H NMR, ^13^C NMR and HR-MS of compounds**


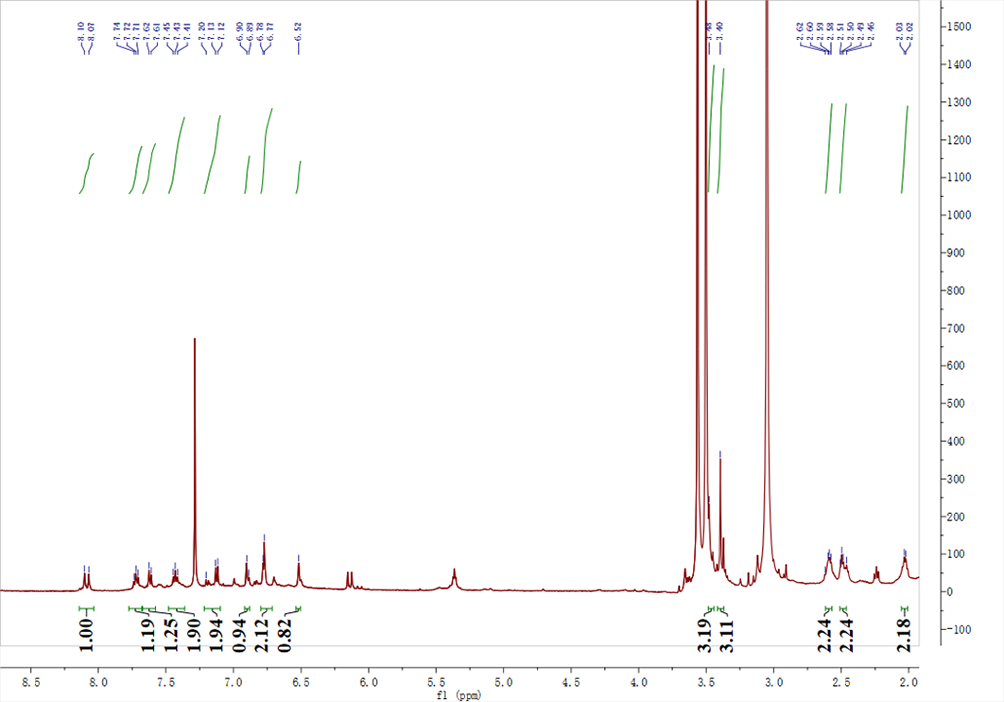


**Figure S7.** ^1^H NMR of HDCX-HClO.


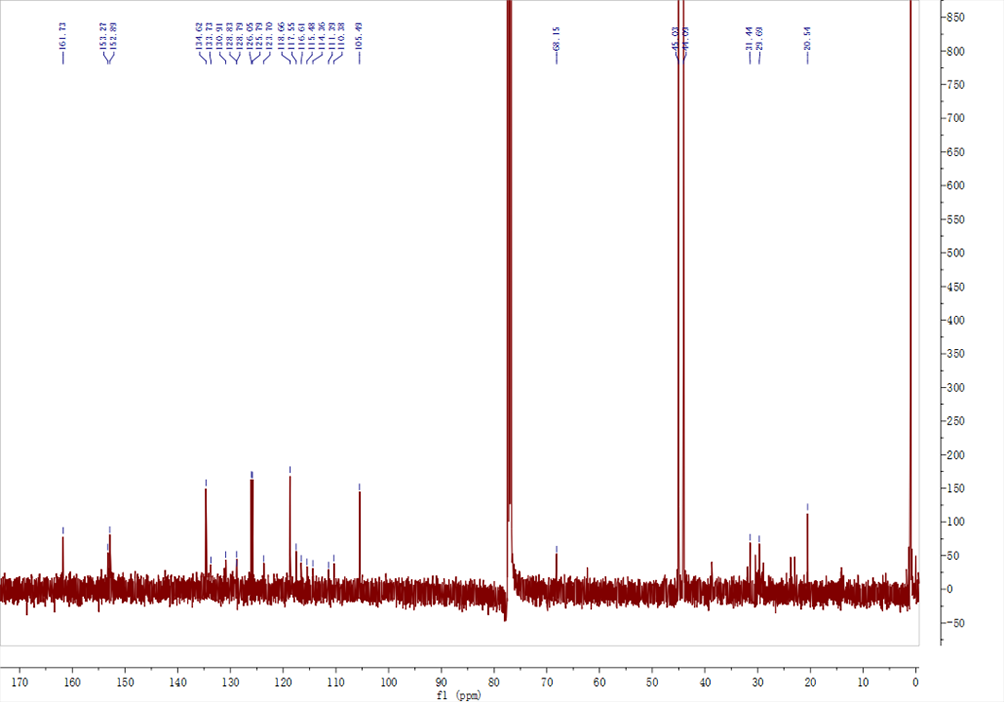


**Figure S8.** ^13^C NMR of HDCX-HClO.


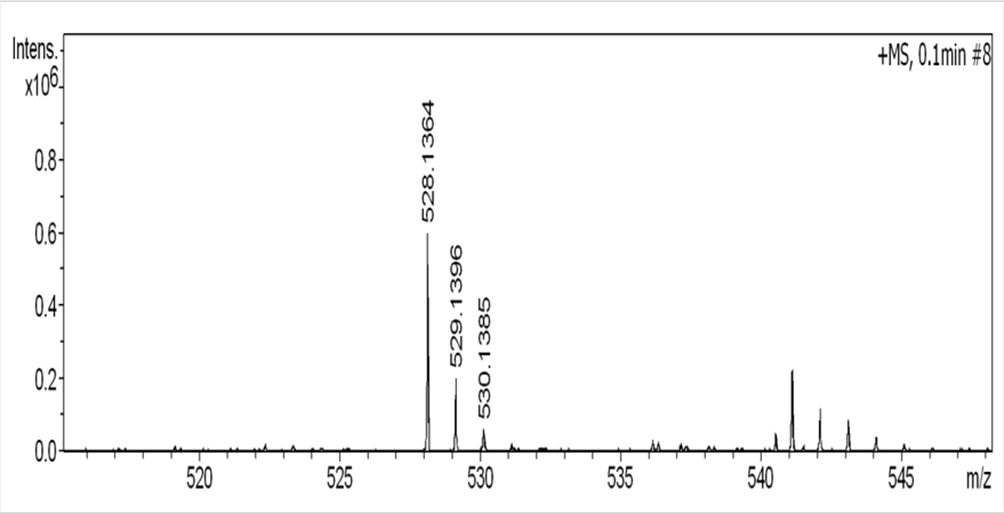


**Figure S9.** HR-MS spectrum of HDCX-HClO.
